# Accent imitation positively affects language attitudes

**DOI:** 10.3389/fpsyg.2013.00280

**Published:** 2013-05-21

**Authors:** Patti Adank, Andrew J. Stewart, Louise Connell, Jeffrey Wood

**Affiliations:** ^1^School of Psychological Sciences, University of ManchesterManchester, UK; ^2^Speech, Hearing and Phonetic Sciences, Division of Psychology and Language Sciences, University College LondonLondon, UK

**Keywords:** imitation, speech, accent, attitudes, stereotypes, perception

## Abstract

People in conversation tend to accommodate the way they speak. It has been assumed that this tendency to imitate each other's speech patterns serves to increase liking between partners in a conversation. Previous experiments examined the effect of perceived social attractiveness on the tendency to imitate someone else's speech and found that vocal imitation increased when perceived attractiveness was higher. The present experiment extends this research by examining the inverse relationship and examines how overt vocal imitation affects attitudes. Participants listened to sentences spoken by two speakers of a regional accent (Glaswegian) of English. They vocally repeated (speaking in their own accent without imitating) the sentences spoken by a Glaswegian speaker, and subsequently imitated sentences spoken by a second Glaswegian speaker (order counterbalanced across participants). After each repeating or imitation session, participants completed a questionnaire probing the speakers' perceived power, competence, and social attractiveness. Imitating had a positive effect on the perceived social attractiveness of the speaker compared to repeating. These results are interpreted in light of Communication Accommodation Theory.

## Introduction

It is well-documented that speakers in conversation have a tendency to converge their speech to their conversation partner's pronunciation patterns (Goldinger, 1998; Namy et al., 2002; Shockley et al., 2004; Pardo, 2006; Pardo et al., 2010; Nielsen, 2011), a phenomenon that is also referred to as *accommodation* or *imitation of speech*. Imitation of speech has been found for intonation (Goldinger, 1998), clarity (Lakin and Chartrand, 2003), speech rate (Giles et al., 1991) regional accent (Delvaux and Soquet, 2007), and speech style (Kappes et al., 2009). This phenomenon seems fairly robust and happens in conversation (Pardo, 2006) but also as a result of mere exposure to speech (Goldinger, 1998; Delvaux and Soquet, 2007). Imitation of speech has received considerable attention in recent years (see Babel, 2011, for an overview) and studies are beginning to map out underlying mechanisms of this behavior in speech production (Pardo, 2006; Babel, 2011).

Imitative behavior during interactions has been shown to increase affiliation and liking between conversation partners (LaFrance and Broadbent, 1976; Chartrand and Bargh, 1999; Dijksterhuis and Bargh, 2002; Van Baaren et al., 2004; Stel et al., 2010). The results of these experiments generally demonstrate that observers have a tendency to imitate their interaction partner's posture and gestures more if they like her or him more. Conversely, observers like their interaction partners more if these partners imitate the observers' posture and gestures. For instance, Stel et al. evaluated the effect of observers' a priori liking of their interaction partner on these observers' tendency to imitate. They asked participants to watch a silent video displaying an actor playing a manager (the target) talking about his work. The target often played with a pen and rubbed his face. A priori liking was manipulated by providing participants with different information. Participants were before watching the video that the manager was entirely honest or dishonest (depending on the a priori liking condition) about the topic he was talking about in the video. They were then asked to fill in a questionnaire to assess a priori liking of the target. Participants were videotaped while observing the video, with one third of the participants instructed to imitate the target, one third explicitly instructed not to imitate the target, and a one third group of participants did not receive any instructions regarding imitation. It was counted how often the participants rubbed their face or played with their pen. The results showed that a priori liking had a positive effect on imitative behavior; participants who had received positive information about the target rubbed their face and played with their pen more often, both in the instructed imitation and the non-instructed imitation conditions. Interestingly, participants who had not been instructed to imitate and participants who had received negative information still imitated the target. This experiment shows a positive relationship between a priori liking and imitative behavior and also demonstrates that imitation occurs even when participants do not show a priori liking.

Another study (Stel et al., 2008) illustrated that the act of imitating a target also affects how the imitator feels toward the target or, more specifically, goals associated with the target. Here, participants were split into two groups and instructed to watch a video of the target describing a charitable organization. In one condition, the participants were instructed to imitate the facial expressions of the target, while the participants in the second condition were instructed not to mimic the target's facial expressions. Subsequently, participants in both conditions were given a questionnaire about the charitable organization and given the opportunity to donate some money if they wished (this money had been provided beforehand to both groups of participants). The results showed that participants who had been instructed to imitate donated more money, which was interpreted as an indication that the imitators had a more pro-social attitude toward the organization than the non-imitators.

Recent work in experimental phonetics similarly points to a relationship between vocal imitation and liking (Babel, 2011). Babel tested how perceived liking affects vocal imitation in a speech production experiment. Liking of the target speaker was measured through a social attractiveness rating on a scale between 1 and 10. Babel found that participants selectively imitated spectral characteristics of only a subset of vowels. Higher imitation rates were found for the low vowels /ae a/ and lower imitation rates for the vowel /i o u/. Also, there was a trend for attractiveness to affect the extent to which participants imitated the target's vowels: (female) participants were more likely to imitate a speaker's vowels if they rated the speaker as more socially attractive. Babel's results are in line with research in social psychology showing that perception of social characteristics (e.g., age, gender, race) of a person performing an action may result in (imitative) behavior congruent with attitudes associated with those characteristics (Bargh et al., 1996; Chen and Bargh, 1997; Dijksterhuis et al., 2000). For instance, Bargh et al. primed participants with attitudes related to old age and subsequently measured the speed with which they walked down a hallway. Participants who had been primed with the old age stereotype walked slower than those who had not been primed (but see Doyen et al., 2012).

Research in social psychology and experimental phonetics thus converges on the notion that a number of factors (such as social attractiveness) can lead to an increase in imitative behavior. However, what is unclear is whether the opposite relationship also holds true: does imitating someone's speech patterns also affect the perceived social attractiveness of that person? If imitative behavior can be shown to affect such attitudes, then this implies that the link between imitation and liking is bidirectional in speech: liking a person results in more imitation of that person's behavior, and imitative behaviors in themselves lead to increased liking of the imitated person.

A recent study presented positive effects of vocal imitation on speech perception (Adank et al., 2010). Adank et al. asked participants to listen to sentences spoken in an unfamiliar accent in background noise in a pre-test phase and repeat these sentences aloud. Subsequently, participants were split into six groups and either received no training, listened to sentences in the unfamiliar accent without speaking, repeated the accented sentences in their own accent, listened to and transcribed the accented sentences, listened to and imitated the accented sentences, or listened to and imitated the accented sentences without being able to hear their own vocalizations. Post-training measures showed participants who imitated the speaker's accent repeated key words in the sentences in higher levels of background noise than participants who had not imitated the accent. Adank et al. demonstrated that vocal imitation of speech affects speech perception by optimizing comprehension of sentences in background noise. Adank et al. thus showed that vocal imitation may aid comprehension of the linguistic message.

The present study aims to establish whether and how vocal imitation affects social attitudes associated with the speaker of this linguistic message. We examined the effect of vocal imitation on attitudes held by listeners toward speakers of a different regional accent than spoken by the listeners themselves. We chose accented speech, as it has already been shown that people spontaneously imitate aspects of their conversation partner's accent (Delvaux and Soquet, 2007). Furthermore, hearing accented speech automatically invokes accent attitudes associated with speakers of the accent (Giles, 1970; Bishop et al., 2005; Coupland and Bishop, 2007).

Here, participants listened to two speakers and overtly imitated the speech patterns for one of these speakers, while they repeated the speech patterns in their own accent for the other speaker. Using a within-subjects design, participants performed these two tasks in counterbalanced order. In one task, they listened to sentences spoken in a regional accent of British English and subsequently repeated these sentences in their own accent, without imitating the accent (repeating phase). Subsequently, they completed a questionnaire probing attitudes related to the speaker's perceived characteristics, including social attractiveness, power, and competence (Bayard et al., 2001). In the other task, participant listened to a different set of sentences spoken by a different speaker of the same regional accent and they were requested to listen to the sentence and repeat it while imitating it as closely as possible (imitating phase). Next, they completed a questionnaire for the second speaker. Participants were thus required to listen to speech from speakers with a regional accent that was different from their own accent. It was decided to select speakers with regional accent as accented speech automatically invokes attitudes associated with its speakers (Lambert et al., 1960). For instance, speakers of standard accents are perceived as more powerful, competent, and having higher social attractiveness than speakers of a regional accent (Giles, 1970; Bishop et al., 2005; Coupland and Bishop, 2007; Grondelaers et al., 2010). If vocal imitation specifically affects listeners' perceived social attractiveness ratings of speakers with a different regional accent, then it is expected that these attitudes are more positive after the experiment's imitation phase.

## Method

### Participants

We tested 52 participants (32 female, 20 male), with an average age of 26.0 years [range 18–55 years, standard deviation (SD) 7.9 years]. All were native speakers from England, with no language impairment or neurological/psychiatric diseases, and with good hearing. We did not monitor the regional background from the participants within England. All participants were undergraduate students enrolled at the University of Manchester. All participants stated to be unfamiliar with Scottish accents in general and Glaswegian specifically when questioned about this during the debriefing session following the experiment. None of them had lived in Scotland or had any close contact with Scottish speakers on an everyday basis. They gave written consent and received course credit, or £5 for participating. The study was approved by the local ethics committee.

### Stimulus materials

The stimulus materials were 96 sentences spoken by two male Glaswegian English (GE) speakers who were 20 and 21 years old at the time the recordings were made. The GE recordings were obtained during the recording session described in Adank et al. (2009). For every speaker, recordings were made of 96 sentences (see Appendix 1) from the Harvard sentences corpus (Egan, 1948; IEEE, 1969). The Harvard sentences are phonetically balanced and semantically meaningful and are frequently used in studies testing speech intelligibility (Rogers et al., 2004).

The GE speakers were recorded in a sound-treated room, using an AKG SE300B microphone (AKG Acoustics, Vienna, Austria), attached to an AKG N6-6E preamplifier, on a Tascam DA P1 DAT recorder (Tascam Div., TEAC Corp., Tokyo, Japan), and transferred directly to hard disk using a Kay Elemetrics DSP sonagraph (Kay Elemetrics, Lincoln Park, NJ). All sentences were saved into individual files at 22,050 Hz. Finally, each sound file was peak-normalized and scaled to 70 dB sound pressure level (SPL), using Praat (Boersma and Weenink, 2003).

We selected GE as we expected that it would be perceived as having low social attractiveness, as it was ranked 29 out of 34 accents of English in terms of its social attractiveness and prestige (Coupland and Bishop, 2007). Coupland and Bishop used ratings based on the responses from the 5010 participants in the Voices project from the British Broadcasting Cooperation's (BBC) that ran throughout 2005 (http://www.bbc.co.uk/voices/). Respondents in the Voices project were fairly evenly distributed across the UK, including Wales (5.6%), Scotland (11.%), Northern Ireland, North/Mid England (39.9%), South-East England (29.1%), South-West England (11.5%).

### Procedure

All participants completed a repeating and an imitation session. The order of these sessions was counterbalanced across participants to avoid task sequence effects; half of the participants completed the imitation session first followed by the repeating session, while the other half imitated first and repeated next. There were 48 sentences per session.

Instructions for the repeating and imitation sessions were taken from Adank et al. (2010). In the repeating session, participants were instructed to listen to the sentence and then to repeat it in their own accent, namely Standard British English. Participants were explicitly instructed not to imitate the speaker's accent. In the imitation session, the procedure was the same as for the repeating session, but participants were instructed to imitate vocally the precise pronunciation of the sentence. If participants repeated the sentence in their own accent, they were instructed to imitate the accent as they heard it spoken. During the repeating task, the experimenter scored the number of correctly repeated content words (see Appendix 1) to ensure that participants understood the sentences. During imitating, the (phonetically naïve) experimenter judged the effort with which participants imitated the speaker's accent on a scale between 1 (very little effort) and 4 (a great deal of effort). The experimenter was instructed to give a score of 1 if they thought that the participant did not attempt to change their speech at all, give a score of 2 if the participant changed their voice, irrespective of whether this was toward the GE accent, and give scores of 3 or 4 if participants attempted to change their voice and managed to replicate aspects of the GE accent. Participants received no feedback other than the experimenter's reminders to keep imitating (in the imitation sessions) or avoid imitating (in the repeating sessions) as described above.

Each participant was tested individually in a quiet room. First, participants repeated 10 familiarization sentences from a male GE speaker whose recordings were not included in the main experiment. Next, they heard 48 sentences over headphones (Sennheiser HD 25 SP) from one of the GE speakers in the repeating session, and the remaining 48 sentences as spoken by the other GE speaker in the imitation session. We included two speakers as it allowed us to evaluate whether the effect of vocal imitation on accent attitudes is general or speaker-specific. As well as counterbalancing for task order, the order of the speakers was counterbalanced across participants, ensuring that speaker 1 was equally often imitated or repeated as speaker 2. If the effect of imitation is speaker-specific, then effects of imitation on social attractiveness ratings differ between speakers.

After each repeating and imitation session, participants were asked to rate their impression of the speaker on 18 personality and voice traits, using a questionnaire (see Appendix 2), which was adapted from Bayard et al. (2001). Bayard et al. developed this questionnaire to examine accent attitudes of New Zealand participants toward different accents of English (New Zealand, Australia and Northern America). The questionnaire consisted of 22 traits: five were voice quality traits (powerful voice, strong voice, educated voice, pleasant voice, attractive voice), 13 were personality traits (controlling, authoritative, dominant, assertive, reliable, intelligent, competent, hardworking, ambitious, cheerful, friendly, warm, humorous), and four status items (occupation, income, social class, education level). The voice quality items and the personality items consisted of Likert-scale questions, asking participants to rate the extent to which the speaker conformed to the trait, while the four status items were set up as open questions. We included only the personality and voice items in the rating scale to allow for answers on a Likert-scale only. In the experiment, participants rated each trait on a scale between 1 and 4 (1: speaker conforms very much to the trait, 4: speaker does not at all conform to the trait).

Participants completed the questionnaire twice: once after the repeating session and once after the imitation session. They were asked to rate their impressions of each speaker. After the experiment, participants were debriefed. Post-experiment debriefing ensured that participants were unaware of the experimental aims and unfamiliar with the Glaswegian accent. The total duration of the experimental procedure (instructions and informed consent procedure, practice session, repeating session, completing questionnaire for the repeating session, imitating session, completing questionnaire for the imitating session, debriefing) was 45 min.

## Results

### Attitudes

Participants correctly repeated 94.8% (SD 3.7%) of four target words per sentence in the repeating phase of the experiment, indicating their understanding of the accented sentences. Furthermore, the average score for the effort judgments obtained during the imitation sessions was 2.2 (SD 0.9), indicating that participants overall were judged to make reasonable efforts when imitating the speaker's accent. Next, the 18 traits were grouped into Power, Competence, and Social Attractiveness attitudes. Following Bayard et al. Six traits were classified as Power attitudes (controlling, authoritative, dominant, powerful voice, strong voice, assertive), six as Competence attitudes (reliable, intelligent, competent, hardworking, educated voice, ambitious), and six as Social Attractiveness attitudes (cheerful, friendly, warm, humorous, attractive voice, pleasant voice). Bayard et al. originally grouped the traits “attractive voice” and “pleasant voice” into a separate “Voice Traits” factor but we decided to pool these factors into the Social Attractiveness attitude to equalize the number of traits per attitude and to ensure that each trait included personality as well as voice traits.

**Figure 1:**
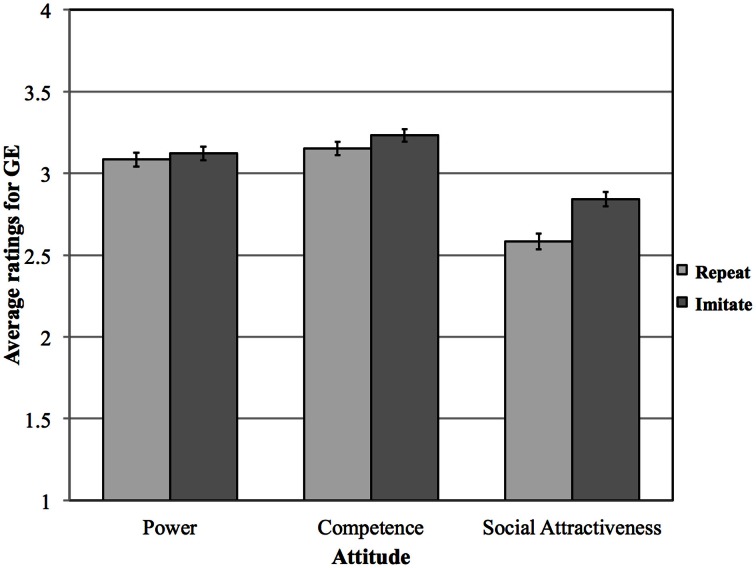
**Average rating scores for Task: Repeat and Imitate, and Attitude: Power, Competence and Social Attractiveness (error bars represent one standard error of the mean) for both GE speakers**.

We recoded all rating scores so that low scores became high scores to make data interpretation more intuitive (i.e., higher scores indicate greater conformity). A 2 (Task: Repeat or Imitate) × 3 (Attitude: Power, Competence, Social Attractiveness) analysis of variance (ANOVA) was conducted on average rating scores. A first main effect was found for Task [*F*_(1, 48)_ = 4.775, *p* < 0.05, partial η^2^ = 0.09]. Rating scores were overall higher after imitating (see Figure 1), indicating that participants found the speakers to conform to the attitudes more after imitating. A second main effect was found for Attitude [*F*_(1.488, 75.89)_ = 21.975, *p* < 0.001, partial η^2^ = 0.3, Huynh-Feldt-corrected for non-sphericity]. Planned *t*-tests showed that the Social Attractiveness Ratings differed from the Competence (*p* < 0.001) and the Power ratings but that the Power and Competence ratings did not differ significantly from each other (*p* = 0.104). Overall, participants judged both the speakers as having better Power and Competences ratings than Social Attractiveness ratings (*p* < 0.001). The main effects for Task and Attitude were qualified by a significant interaction [*F*_(1.826, 93.126)_ = 3.371, *p* < 0.05, partial η^2^ = 0.06], indicating that the effects of task affected the three attitudes differently. *Post-hoc* tests showed that only Social Attractiveness judgments were significantly more positive (*p* = 0.007), i.e., the speaker was judged to conform more to the trait, after imitation. This indicated that participants rated the speakers as having higher Social Attractiveness after imitation sessions than after repeating sessions.

Finally, we calculated difference scores between ratings of the imitation and the repeating phases and we correlated these difference scores with the individual effort scores obtained during the imitation phase of the experiment. Imitation effort scores did not correlate significantly with Social Attractiveness, Power, or Competence difference scores.

### Individual traits

**Figure 2:**
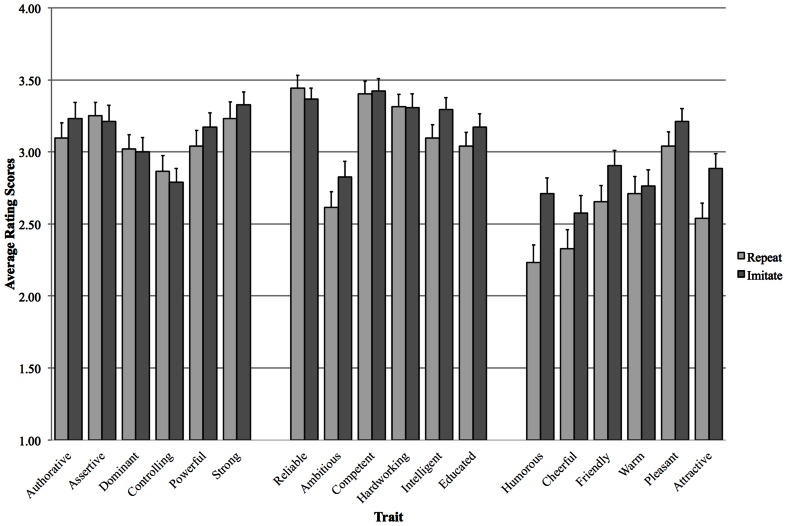
**Average rating scores for Task: Repeat and Imitate, for all 18 traits (error bars represent one standard error of the mean) for both GE speakers**.

Figure 2 shows the average ratings for the individual traits. We ran planned *t*-tests between the ratings obtained after imitating and repeating for each trait. The planned *t*-tests for the Power traits (Authoritative, Dominant, Assertive, Controlling, Powerful voice, Strong voice) showed no significant differences at *p* < 0.05. No differences were found either for the Competence traits (Reliable, Ambitious, Competent, Intelligent, Hardworking, Educated voice). However, effects were found for three pairs for the Social Attractiveness traits (Humorous, Cheerful, Friendly, Warm, Pleasant voice, Attractive voice). Participants rated the speakers as more humorous after imitating [*t*_(51)_ = −3.468, *p* = 0.001], as being more friendly [*t*_(51)_ = −2.095, *p* = 0.041] and as having a more attractive voice [*t*_(51)_ = −3.163, *p* = 0.003].

## Discussion

We aimed to establish whether vocal imitation of sentences spoken in an unfamiliar accent positively affected social attractiveness ratings associated with the speaker of these sentences. The results confirm our hypothesis, as the ratings of a GE speaker's social attractiveness were more positive after the participant had vocally imitated sentences produced by that speaker. Furthermore, the results showed that the positive effects were found only for the Social Attractiveness ratings and not for the Competence and Power ratings. This pattern in the results allows us to rule out alternative explanations for the effect of imitation, such as increased attention or more effortful processing during the imitation phases of the experiment. It seems unlikely that increased attention or more effortful processing would specifically affect Social Attractiveness, but not Power and Competence ratings. Nevertheless, imitation effort ratings did not correlate with the difference scores for Social Attractiveness, indicating that participants who were judged to exert greater effort did not show a tendency to change their judgments more than those who were judged to have exerted less effort during imitating.

The pattern in the ratings of the three attitudes differed from earlier studies on attitudes on English accents (Bishop et al., 2005; Coupland and Bishop, 2007). We found less positive ratings for Social Attractiveness than for Power and Competence. It is unclear why this is the case, but this could be due to the fact that we tested a relatively select group of participants, namely young undergraduate students from England only, while the listeners in the original BBC project described in Bishop et al. (2005), Coupland and Bishop (2007) originated from all over the UK and included younger and older participant groups and was not restricted to university students alone. Speakers of specific regional varieties of British English may exhibit different patterns in their attitudes toward specific accents.

Finally, the effect of imitation was only found for Social Attractiveness but not for Competence and Power. The effect of imitating on Social Attractiveness was driven by the traits Humorous, Friendly and Attractive Voice. It is possible that the act of imitating another's accent makes the speaker part of participants' social in-group in a way that mere repetition does not. Since people are more positively biased toward people in their in-group than those outside (Brewer, 1979), such *in-group favoritism* could make the speaker seem more subjectively pleasant while having little effect on power and competence attitudes, which may be less flexible, possibly due to lower susceptibility to generalized attitudes toward unfamiliar accents and speakers of those accents.

### Limitations

It should be noted that the effect of imitation on the Social Attractiveness ratings does not necessarily imply that participants' attitudes toward the Glaswegian accent *per se* have changed. Rather, it may be that the attitudes toward the GE speaker who was imitated have changed. Therefore, imitating the speech of speakers who speak in a relatively unfamiliar way may lead to a more positive appreciation of these speakers' social attractiveness characteristics. However, note that it is not easy to isolate the speaker from the accent. Evaluating to which extent the attitudes toward an individual versus her or his group characteristics (the regional accent) may not be straightforward, as speaker and accent are inherently confounded. A solution would be to run the experiment using a matched-guise speaker (Lambert et al., 1960), i.e., someone who can speak two accents. See for example Evans and Iverson (2004), who used speech from a speaker who spoke Standard Southern British English as well as a Northern British accent. Using a matched-guise speaker would open up possibilities to tease apart the effect of imitating an individual versus imitating an accent.

Also, we cannot entirely exclude the possibility that the positive effect of imitation on the Social Attractiveness judgments is due to the instruction to explicitly *not* imitate in the repeating task. One way to determine whether the effect on Social Attractiveness is entirely due to imitation and not to the suppression of imitation in the repeating sessions would be to include a control condition in which participants did not receive any explicit instructions regarding imitation. However, such a control condition would not be feasible within the current within-subject design with task order (and speaker) counterbalanced across participants, as was the case in the present study.

Finally, recent studies measuring the effect of attitudes toward a speaker on vocal imitation used acoustic measures (Babel, 2011) or perceptual similarity judgments (Namy et al., 2002; Pardo, 2006; Pardo et al., 2010, 2012) to access the extent to which participants change their speech. For instance, Babel (2011) used measurements of the first two formant center frequencies of the vowels in the words her participants were required to shadow. Pardo et al. (2010) used perceptual measures of phonetic convergence in her conversational design. She asked a group of phonetically naïve listeners to judge the similarity between utterances of two conversation partners recorded before, during and after both took part in a goal-directed task (a map-task in which specific items were name by both partners) in an ABX task. Measures of perceived convergence were then computed by scoring the percent of trials on which a specific item produced after the map-task was judged to be more similar to this item as produced in map task item than it was to the item produced prior to the map task. The present study did not investigate the effect of perceived aspects of the target on vocal imitation, but the effect of vocal imitation on perceived speaker characteristics. However, the study could have benefited from the use of more sophisticated—and objective—measures of vocal imitation performance, such as used in Babel (2011). However, a disadvantage of using acoustic measurements is that data collection and analysis can be extremely time-consuming and that the effect of vocal imitation may not be fully captured using only measures of vowel quality. It would be interesting to pattern-matching methods also used to measure accent similarity, such as the program ACCDIST (Huckvale, 2004) and apply this to individual pairings of the imitator's and the target's sentences. For instance, Pinet et al. (2011) used ACCDIST successfully to establish accent differences between French-English bilinguals and British English. A method such as ACCDIST could be used to provide a more fine grained measure of the extent to which the participants (a) changed their speech between repeating and imitating and (b) to which extent the participant's utterances in the imitation sessions resemble the target speaker's utterances. Such an objective acoustic measure would be an improvement over the effort judgments used in the present experiment. Nevertheless, the current effort judgments from the experimenter in the imitating phases give at the very least an impression of the extent to which the participant attempted to imitate the sentences in the imitation session, but their relevance should not be overstated.

### Communication accommodation theory

Phonetic convergence, or the process by which conversation patterns change the acoustic characteristics toward a common target, has been accounted for using Communication Accommodation Theory (Giles et al., 1991; Shepard et al., 2001). Communication Accommodation Theory accounts for phonetic convergence and divergence by exploring the various explanations of processes through which individuals decrease or increase the social distance between themselves and others through verbal and non-verbal behaviors. Phonetic convergence, for instance as demonstrated in Pardo et al. (2012) and Pardo et al. (2010), is seen as one of the mechanisms through which individuals decrease the social distance. This decrease may have the effect of making the interaction flow more smoothly (Pardo et al., 2012). The present results showed that overt changing of an individual's speech toward a target positively affects feeling of sociability toward that target. This process may thus represent another mechanism through which individuals decrease the social distance. This notion is rather speculative, as we did not test individuals in conversation. However, it would be interesting to investigate this possibility in a dyadic design in which conversation partners' mutual liking is manipulated. Liking one's conversation partner could then make one imitate that partner more, in analogy with Stel et al. (2010), and imitating could in turn increase liking more. Furthermore, it could also be the case that the link between imitation and liking also serves to increase social distance. In this scenario, disliking someone may lead conversation partners to imitate less which in turn then leads to even less liking, leading ultimately to an increase of social distance.

### Conclusion

In sum, the present research demonstrates that vocal imitating of speech positively alters attitudes about the speaker's perceived Social Attractiveness. These results are in line with previous social psychological studies that found a positive effect of imitation on affiliation for the interaction partner being imitated (LaFrance and Broadbent, 1976), as well as for the individual imitating his or her interaction partner (Stel and Vonk, 2010). Finally, it has already been shown that vocal imitation enhances action perception under ambiguous/noisy listening conditions (Adank et al., 2010), or that vocal imitation improves understanding of the speaker's *message*. Our results indicate that imitation effects extend to evaluation of the speaker's social characteristics.

### Conflict of interest statement

The authors declare that the research was conducted in the absence of any commercial or financial relationships that could be construed as a potential conflict of interest.

## Appendix 1

Sentence materials used in the experiment. The 10 familiarization sentences were presented in the order listed across all participants, while the 96 test sentences were randomized across participants.

**Table:**

	**Familiarization sentences**	**Key words**
1	Plead with the lawyer to fight the lost cause	Plead lawyer lost cause
2	Pile the coal high in the shed corner	Pile coal shed corner
3	Be sure to set the lamp firmly in the hole	Sure set lamp hole
4	We don't like to admit our small faults	Admit our small faults
5	The pup jerked the leash as he saw a feline shape	Pup leash feline shape
6	The hail pattered on the burnt brown grass	Hail pattered burnt grass
7	Open your book to the first page	Open book first page
8	The long journey home took a year	Long journey took year
9	Small children came to see him	Small children see him
10	A severe storm tore down the barn	Severe storm down barn
	**Test sentences**	**Key words**
1	Glue the sheet to the dark blue background	Glue sheet dark background
2	Rice is often served in round bowls	Rice served round bowls
3	It's easy to tell the depth of a well	Easy tell depth well
4	A large size in stockings is hard to sell	Large size stockings sell
5	Four hours of steady work faced us	Four hours steady work
6	The salt breeze came across from the sea	Salt breeze came sea
7	The girl at the booth sold fifty bonds	Girl booth fifty bonds
8	The swan dive was far short of perfect	Swan dive short perfect
9	Hoist the load to your left shoulder	Hoist load left shoulder
10	The fish twisted and turned on the bent hook	Fish twisted turned hook
11	Wipe the grease off his dirty face	Wipe grease dirty face
12	The stray cat gave birth to kittens	Stray cat birth kittens
13	The ship was torn apart on the sharp reef	Ship torn apart reef
14	Sickness kept him home the third week	Sickness kept home week
15	The crooked maze failed to fool the mouse	Crooked maze fool mouse
16	The show was a flop from the very start	Show flop very start
17	March the soldiers past the next hill	March soldiers past hill
18	The set of china hit the floor with a	China hit floor crash
19	A tame squirrel makes a nice pet	Tame squirrel nice pet
20	The clock struck to mark the third period	Clock struck mark period
21	Cut the pie into large parts	Cut pie large parts
22	He lay prone and hardly moved a limb	Lay prone moved limb
23	Bail the boat to stop it from sinking	Bail boat stop sinking
24	The term ended in late June that year	Term ended June year
25	The bill was paid every third week	Bill paid third week
26	The ripe taste of cheese improves with age	Taste cheese improves age
27	Split the log with a quick, sharp blow	Split log sharp blow
28	Weave the carpet on the right hand side	Weave carpet hand side
29	Type out three lists of orders	Type three lists orders
30	Feel the heat of the weak dying flame	Feel heat dying flame
31	Mud was splattered on the front of his white shirt	Mud spattered front shirt
32	The urge to write short stories is rare	Urge short stories rare
33	The jacket hung on the back of the wide chair	Jacket hung back chair
34	Torn scraps littered the stone floor	Torn scraps littered floor
35	Fairy tales should be fun to write	Fairy tales fun write
36	Acid burns holes in wool cloth	Acid burns holes cloth
37	Eight miles of woodland burned to waste	Eight miles woodland burned
38	We admire and love a good cook	Admire love good cook
39	He carved a head from the round block of marble	Carved head block marble
40	She has a smart way of wearing clothes	Smart way wearing clothes
41	Corn cobs can be used to kindle a fire	Corn cobs used kindle
42	Bring your best compass to the third class	Bring best compass class
43	The brown house was on fire to the attic	Brown house fire attic
44	The lure is used to catch trout and flounder	Lure catch trout flounder
45	The loss of the second ship was hard to take	Loss second hard take
46	Live wires should be kept covered	Live wires kept covered
47	The large house had hot water taps	Large house water taps
48	Write at once or you might forget it	Write once may forget
49	The lamp shone with a steady green flame	Lamp shone steady flame
50	Rake the rubbish up and then burn it	Rake rubbish up burn
51	They are pushed back each time they attack	Pushed back time attack
52	Some ads serve to cheat buyers	Ads serve cheat buyers
53	The birch looked stark white and lonesome	Birch looked stark lonesome
54	Look in the corner to find the tan shirt	Corner find tan shirt
55	Nine men were hired to dig the ruins	Nine hired dig ruins
56	The flint sputtered and lit a pine torch	Flint sputtered pine torch
57	A cloud of dust stung his tender eyes	Cloud dust stung eyes
58	The old pan was covered with hard fudge	Pan covered hard fudge
59	Watch the log float in the wide river	Watch log float river
60	The barrel of beer was a brew of malt	Barrel beer brew malt
61	The peace league met to discuss their plans	Peace league discuss plans
62	Boards will warp unless kept dry	Boards warp unless dry
63	Let it burn, it gives us warmth and comfort	Burn gives warmth comfort
64	Tack the strip of carpet to the worn floor	Strip carpet worn floor
65	The man went to the woods to gather sticks	Man woods gather sticks
66	The dirt piles were lines along the road	Dirt piles lines road
67	The logs fell and tumbled into the clear stream	Logs fell tumbled stream
68	Soap can wash most dirt away	Soap wash dirt away
69	Fake stones shine but cost little	Fake stones shine little
70	The square peg will settle in the round hole	Square peg settle hole
71	Heave the line over the port side	Heave line port side
72	A list of names is carved around the base	List names around base
73	Grace makes up for lack of beauty	Grace makes lack beauty
74	Nudge gently but wake her now	Nudge gently wake her
75	Bottles hold four kinds of rum	Bottles four kinds rum
76	The man wore a feather in his felt hat	Man feather felt hat
77	Turn out the lantern which gives us light	Turn lantern gives light
78	Birth and death mark the limits of life	Birth death mark limits
79	The chair looked strong but had no bottom	Chair looked strong bottom
80	Five years he lived with a shaggy dog	Five years lived dog
81	He offered proof in the form of a large	Offered proof large chart
82	The three story house was built of stone	Storey house built stone
83	We like to see clear weather	Like see clear weather
84	The door was barred, locked, and bolted as well	Door barred locked bolted
85	Ripe pears are fit for a queen's table	Ripe pears queen's table
86	The vast space stretched into the far distance	Vast space stretched distance
87	A rich farm is rare in this sandy waste	Farm rare sandy waste
88	Hurdle the pit with the aid of a long	Hurdle pit aid pole
89	The square wooden crate was packed to be shipped	Square wooden crate shipped
90	Down that road is the way to the grain	Down road grain farmer
91	A toad and a frog are hard to tell	Toad frog tell apart
92	A round hole was drilled through the thin board	Round hole drilled board
93	Prod the old mule with a crooked stick	Prod mule crooked stick
94	Dull stories make her laugh	Dull stories make laugh
95	He lent his coat to the tall gaunt stranger	Lent coat tall stranger
96	The duke left the park in a silver coach	Duke left park coach

## Appendix 2

### Questionnaire used after imitation and repeat tasks

Please rate your impressions of the speaker's personality. Circle 1 if you think the speaker's personality does conform very much to the trait, and choose 4 if your think the speaker's personality does not conform at all to the trait.

**Table:**

	**Trait**	**Very**	**A bit**	**Not very much**	**Not at all**
1	Reliable	1	2	3	4
2	Ambitious	1	2	3	4
3	Humorous	1	2	3	4
4	Authoritative	1	2	3	4
5	Competent	1	2	3	4
6	Cheerful	1	2	3	4
7	Friendly	1	2	3	4
8	Dominant	1	2	3	4
9	Intelligent	1	2	3	4
10	Assertive	1	2	3	4
11	Controlling	1	2	3	4
12	Warm	1	2	3	4
13	Hardworking	1	2	3	4

### Voice

Please rate your impressions of the speaker's voice. Circle 1 if you think the speaker's voice does conform very much to the trait, and choose 4 if your think the speaker's voice does not conform at all to the trait.

**Table:**

	**Trait**	**Very**	**A bit**	**Not very much**	**Not at all**
1	Powerful	1	2	3	4
2	Strong	1	2	3	4
3	Educated	1	2	3	4
4	Pleasant	1	2	3	4
5	Attractive	1	2	3	4
